# Developing CAR-T/NK cells that target EphA2 for non-small cell lung cancer treatment

**DOI:** 10.3389/fimmu.2025.1448438

**Published:** 2025-03-13

**Authors:** Seok Min Kim, Soo Yun Lee, Seo In Kim, Ji Yeong Bae, Jin Tae Hong, Seona Jo, Ji Hyun Kim, Hyo-Young Chung, Tae-Don Kim

**Affiliations:** ^1^ Center for Gene & Cell Therapy, Korea Research Institute of Bioscience and Biotechnology (KRIBB), Daejeon, Republic of Korea; ^2^ New Drug Development Center, Osong Medical Innovation Foundation, Cheongju-si, Chungbuk, Republic of Korea; ^3^ College of Pharmacy and Medical Research Center, Chungbuk National University, Cheongju-si, Chungbuk, Republic of Korea; ^4^ Department of Functional Genomics, KRIBB School of Bioscience, Korea University of Science and Technology (UST), Daejeon, Republic of Korea

**Keywords:** cell therapy, chimeric antigen receptor (CAR), CAR-T cell, CAR-NK cell, erythropoietin-producing hepatocellular carcinoma A2 (EphA2), non-small cell lung cancer (NSCLC)

## Abstract

**Introduction:**

Chimeric antigen receptor (CAR) immunotherapy has revolutionized anticancer therapy, as it accurately targets cancer cells by recognizing specific antigens expressed in cancer cells. This innovative therapeutic strategy has attracted considerable attention. However, few therapeutics are available for treating non-small cell lung cancer (NSCLC), which accounts for most lung cancer cases and is one of the deadliest cancers with low survival rates.

**Methods:**

In this study, we developed a new antibody targeting erythropoietin-producing hepatocellular carcinoma A2 (EphA2), which is highly expressed in NSCLC, and established CAR-T/ natural killer (NK) immune cells to verify its potential for immune cell therapy. The killing capacity, cytokine secretion and solid tumor growth inhibition of EphA2 CAR-T/NK cells were compared to normal T/NK cells.

**Results:**

EphA2 CAR-T cells demonstrated superior killing capacity, enhanced cytokine secretion, and significant solid tumor growth inhibition. Additionally, they exhibited improved tumor infiltration in lung cancer models compared to normal T cells. The anticancer efficacy of the developed EphA2 CAR-NK cells was also confirmed, showcasing their potential as robust candidates for immune cell therapy.

**Discussion:**

The findings of this study highlight the potential of CAR-T/NK cell therapy targeting EphA2 as an effective treatment for lung cancer, particularly NSCLC with high EphA2 expression. By leveraging the specific targeting capabilities of CAR-T cells and the unique properties of CAR-NK cells, this approach provides a promising therapeutic strategy to address the unmet needs in NSCLC treatment.

## 1 Introduction

Although the survival rate of patients with cancer has increased since the 2000s, lung cancer has been deadly, with a survival rate of 28.2% over the past five years, which sharply declines to 6.1% when distant metastasis is present (1). Non-small cell lung cancer (NSCLC), which accounts for most lung cancer cases, has increased fatality, with a survival rate of approximately 4% over five years and a continuous increase in mortality (2, 3). Several cancer treatments, such as surgery, radiation, chemotherapy, and antibody therapy, have been developed over the past several years. However, these methods often have side effects that impact both cancerous and normal cells.

Consequently, alternative treatments, such as immunotherapy have garnered significant interest. Recently, chimeric antigen receptor (CAR) immunotherapy has gained attention as an innovative approach that minimizes side effects by accurately targeting cancer cells and recognizing specific protein antigens expressed on them (4). CAR-T cells are adaptive T cell therapies for personalized treatment (5). In particular, CAR-T cells targeting CD19 have shown successful treatment outcomes in patients with B-cell ALL. After the Food and Drug Administration approved CAR-T cell treatment, its high therapeutic capacity was recognized (6). CAR-T cells effectively destroy cancer cells, reduce damage to normal cells, and secrete sufficient cytokines, demonstrating their high efficacy and safety. However, CAR-T therapy has several disadvantages such as cytokine release syndrome (CRS), neurotoxicity, and limited effectiveness in solid cancers (4).

In addition to CAR immunotherapy using T cells, strategies utilizing various immune cell types, particularly natural killer (NK) cells, have been used extensively (7). NK cells are used in cell therapy because they are characterized by selective cytotoxicity that distinguishes between normal and cancerous cells (8). As CAR-NK cells are not activated by major histocompatibility complex signaling, the risk of alloreactivity is reduced, enabling mass production. These cells are always prepared as ready-made products for injection into patients at any time. It can also be produced from various sources, such as NK92 cells, peripheral blood mononuclear cells, and induced pluripotent stem cells. In addition, CRS and neurotoxicity are less likely to occur (9). However, CAR-NK cell therapy did not have a significant effect on solid cancers (10).

Selecting an appropriate cancer antigen that maximizes treatment effectiveness and safety is crucial for developing effective cancer therapies using CAR-T and CAR-NK cells. Erythropoietin-producing hepatocellular carcinoma A2 (EphA2), a member of the EPH family of receptor tyrosine kinases, is widely expressed in various cancers and plays a pivotal role in tumor development and progression (11). EphA2 facilitates malignant progression by mediating interactions between tumor cells and their microenvironment, including endothelial and immune cells (12), and is associated with poor clinical prognosis, particularly in non-small cell lung cancer (NSCLC) (13, 14). EphA2 is overexpressed in more than 90% of NSCLC tissues, where it correlates with poor survival rates and drives tumor growth, metastasis, and survival through mechanisms such as S897 phosphorylation and ERK1/2 activation (13, 14). Suppression of EphA2 expression has been shown to harden spheroids and significantly reduce the cancer area, underscoring its role in metastasis (15). As a tumor-associated antigen, EphA2 has been extensively studied over the past 25 years, establishing its status as a promising molecular target for therapeutic development and clinical translation in NSCLC and other cancers (16–18).

Therefore, to develop new treatments for NSCLC, our study aimed to identify novel antibodies targeting EphA2 and incorporate them into CAR, with the potential to develop CAR-T/NK cell immunotherapy a treatment for NSCLC. We engineered CAR-T cells targeting tumor-specific EphA2 antigens and confirmed that the tumor-killing activity and pro-inflammatory cytokine production in A549 cells were significantly higher than those in normal T cells. We also confirmed that EphA2 CAR-T cells exhibited strong anticancer effects against solid tumors in a xenograft mouse model. Similar to CAR-T cells, we engineered CAR-NK92 cells targeting EphA2 and showed that EphA2 CAR-NK92 cells exhibited high anticancer efficacy against H460 cells *in vitro* and *in vivo*. Thus, our results suggest that CAR-T/NK cells targeting EphA2 may be an efficient immune cell therapy for lung cancer, particularly NSCLC, with increased EphA2 expression.

## 2 Materials and methods

### 2.1 Cell lines and culturing condition

Human non-small cell lung cancer (NSCLC) cell lines, A549 & H460 and human leukemia cell line, K562 were purchased from the American Type Culture Collection (ATCC, USAA549). A549 cells expressing GFP or luciferase were generated by the transduction of each genome bearing lentivirus vector, followed by puromycin selection. A549-luciferase was transformed by the luciferase gene-expressed lentiviral system. The cell line demonstrates the high level of bioluminescence signal. A549 cells in Dulbecco’s modified Eagle’s medium (Gibco, USA) and K562 & NCI-H460 cells in Roswell Memorial Institute medium-1640 (Gibco, USA) were maintained at 37°C with 5% CO_2_. The NK92 (human natural killer cell line, ATCC^®^CRL-2407™) were purchased from American Type Culture Collection (Manassas, VA, USA). The NK-92 cells were grown in alpha minimum essential medium which contained 12.5% fetal bovine serum (FBS), and 12.5% horse serum. To prepare the complete growth medium, the following components were added to the medium prior to use: 0.2 mM inositol, 0.1 mM 2-mercaptoethanol, 0.02 mM folic acid, and 200 U/ml recombinant IL-2 (Peprotech, Cat. 200-02).

### 2.2 Analysis of anti-EphA2 scFv sensitivity

ELISA: A 96-well ELISA plate was prepared by adding 30 µL of antigen protein (2 µg/mL in PBS) or control (PBS only) to each well, followed by incubation at 4°C for 16 h. Subsequently, 150 µL of MPBS (PBS containing 5% skim milk) was added to each well to block nonspecific binding and incubated at room temperature (RT) for 1 h. Serial dilutions of the phage were prepared in PBS. After removing the blocking solution, 30 µL of each phage dilution was added to the wells and incubated at 37°C for 1 h. The wells were washed four times with PBST, followed by incubation with anti-M13 antibody conjugated with HRP (1:5000; Thermo Fisher Scientific, USA) at 37°C for 1 h. Afterward, the wells were washed four more times with PBST, and TMB substrate (Sigma, Japan) was added to develop color for 4 min. The reaction was stopped, and absorbance was measured at 450 nm. The resulting absorbance data were analyzed using a 4-parameter logistic regression to generate a graph and calculate the EC50 value.

FACS Analysis: The assay was conducted in 96-well plates, with approximately 10^6^ cells per well. Phages (10¹¹ pfu) were added to the cells and incubated at 4°C for 1 h to allow binding. After the final washing step with PBS (performed twice), the cells were resuspended in PBS and stained with anti-M13-FITC antibody at 4°C for 1 h. Following staining, the cells were washed again, resuspended in PBS, and fluorescence was measured using flow cytometry.

### 2.3 Lentiviral vector production and titration

EphA2 CAR gene bearing lentiviral vector was produced using 3^rd^ generation lentiviral vector system (Addgene, USA). Briefly, 6 × 10^6^ 293T cells were seeded at 10cm culture plate to achieve ~80% confluency at the time of transfection. The next day, cells were transfected using Lipofectamine 3000 (Thermofisher, USA) with lentiviral vector plasmids as follows: 5.5 µg of pLV-EphA2 CAR, 3.5 µg of pMDLg/pRRE, 1.5 µg of pRSV-Rev and 2 µg of pMD2.G. At 48 hours after transfection, viral vector containing supernatant was harvested and clarified by centrifugation to remove cell debris, filtration through 0.45 µM filter. Lentiviral vectors were stored at -80°C before titration.

### 2.4 Generation of T cells expressing CAR

Human peripheral blood mononuclear cells (Lonza, Switzerland) were activated with the T cell TransAct (Miltenyi Biotec, Germany) on 24-well plates in RPMI 1640 medium supplemented 200 IU/ml rhIL-2 (Peprotech, USA) at 5% CO_2,_ 37°C. At 1 day after activation, cells were transduced with Δ Ecto or EphA2 CAR lentivirus at a multiplicity of infection (MOI) of 5 in presence of 10 µg/mL protamine sulfate and centrifuged for 90min at 300 g, 32°C. Cells were kept in 10% FBS containing RPMI 1640 medium with 200 IU/ml rhIL-2 and half of the media was exchanged with fresh one at every 2 days. Transduced cells were expanded for up to 14 days.

### 2.5 Generation of NK cells expressing CAR

Δ Ecto or EphA2 CAR lentivirus was infected into NK92 cells at a Multiplicity of Infection (MOI) of 30 by spinoculation method (360 g, 90min, room temperature). The infected NK92 cells were incubated at 37°C, 5% CO2 for 5 hours, then changed to fresh medium and were cultivated by treating with puromycin at a concentration of 3 ug/ml in order to screen properly infected NK92 cells after 3 days. As a control, uninfected NK92 cells were also treated with puromycin and cultivated continued using a puromycin-treated medium until all NK92 cells in control were killed by puromycin. Infected NK92 cells were picked at the point when all the natural killer cells in the control group were killed, and experiments were performed.

### 2.6 Flow cytometric analysis

To analyze EphA2 CAR protein expression and T cell subset population, CAR-T cells were harvested on day 12 after transduction and stained with the following fluorochrome-conjugated anti-human antibodies: EphA2 (FAB3035G, Abcam), CD3 (563423, BD Bioscience), CD4 (560345, BD Bioscience), CD8 (557834, BD Bioscience), Recombinant EphA2 protein (ATGP3378, NKMAX), His-coupled FITC (ab1206, Abcam). The antibodies were used at 1 µg per sample for 20 min at 4°C in the dark. CD107a expression in EphA2 CAR-NK92 cells was evaluated after coculture with target cells at a 1:1 E:T ratio for 4 hours. Cells were washed with cold FACS buffer and incubated with the following fluorochrome-conjugated mouse anti-human antibodies: CD56 (555518, BD Bioscience), CD107a (555801, BD Bioscience), Myc-Tag (9B11) (3739S, Cell signaling), and hEphA2(FAB3035G, RnD systems). The antibodies were used at 1:100 dilution for 30 min at 4°C in the dark. All data were acquired on a BD FACS Canto II cytometer (BD Bioscience, USA), and analyzed using the FlowJo software (FlowJo LLC, USA).

### 2.7 Real-time cytotoxicity assay

CAR-T cell mediated cytotoxicity was examined with Incucyte Zoom (Essen Bioscience, USA). The A549-GFP cells (5 × 10^3^ cells per well) were placed into a 96-well round-bottom plate and cultured for 24 hours, and then Δ Ecto and EphA2 CAR-T cells were added into A549-GFP cells at E:T ratio 1:1. Data were measured at every 4 hours for 72 hours. Specific target cell lysis was calculated according to the following formula: GFP intensity of wells with CAR-T cells/GFP intensity of wells without CAR-T cells.

### 2.8 Calcein-AM release assay

NK cell–mediated cytotoxicity was examined with a standard 4-hour calcein-AM cytotoxicity assay. In brief, target cells were incubated with 5ug/ml calcein-AM for 1hour. Calcein-AM stained target cells (1 × 10^4^cells per well) were placed into a 96-well round-bottom plate in triplicate and cocultured with Δ Ecto or EphA2 CAR-NK92 cells at E:T ratios ranging from 5:1, 1:1, 0.5:1 for 4hours. Calcein release from lysed target cells by Δ Ecto or EphA2 CAR-NK92 was measured using a fluorescence plate reader (Spectra Max i3x, excitation filter, 485nm; emission filter, 535nm). The percentage of specific lysis was calculated according to the following formula: (Experimental release-Spontaneous release)/(Maximum release –Spontaneous release) × 100.

Spontaneous release refers to calcein released from target cells in complete medium alone, whereas maximum release refers to calcein release from target cells in complete medium containing 2% Triton X-100.

### 2.9 Cytokine release assay

Enzyme-linked immunosorbent assay (ELISA) was performed to quantify cytokines released by CAR-T or CAR-NK cells. Δ Ecto or EphA2 CAR-T cells were cocultured with A549 for 16 hours at the different effector to target cell ratios (E:T) of 2:1, 1:1, 0.5:1, 0.25:1. The supernatants were gathered and ELISA measurements were conducted to quantitate expressed Interferon-γ (IFN-γ), tumor necrosis factor-α (TNF-α), and Granzyme B. In case of EphA2 CAR-NK92, secreted IFN-γ level was measured using the medium of EphA2 CAR- NK92 cells cocultured with target cells (H460, MDA-MB-231 and K562) at a 1:1 E:T ratio for 16 hours.

### 2.10 Antitumor activity in a xenograft mouse model

All animal experiments were approved by the Institutional Animal Care and Use Committee (IACUC). To make A549 xenograft model, NOD/Shi-scid, IL2Rγnull (NOG) mice were treated with 1 × 10^6^ A549-Luc cells per mice subcutaneously. When the tumor size had reached approximately 200 mm^3^, 24 mice were separated into three groups randomly (8 mice per group: 5 mice for the estimation of antitumor and 3 mice for the tumor infiltration lymphocyte). The mice were injected with 5 × 10^6^ Δ Ecto or EphA2 CAR-T cells intravenously. Tumor size was measured twice a week with calipers and calculated using the formula length × width^2^ × 0.5. Peripheral blood was extracted every two weeks to confirm T cell proliferation in mice. Extracted cells from mice’s blood were stained with the same as above FACS antibodies. On day 45 after infusion, mice were sacrificed and tumors were harvested. All mice were imaged by the Xenogen IVIS imaging system once a week for up to 45 days. Bioluminescence imaging was performed using the IVIS-Lumina II imaging system (PerkinElmer). Mice were anesthetized, and then given an intraperitoneal (i.p.) injection of D-luciferin (150mg/kg body weight). After 10 min, images were analyzed using Living Image software (PerkinElmer), and data were presented as the total flux(photons/s). Images were analyzed by Living Image 4.3 software. For the H460 xenograft mouse model, five-week-old male BALB/c nude mice were purchased from the Saeronbio (Republic of Korea). 3 × 10^6^ H460 cells were injected subcutaneously in flanks of mice in a solution of 50% Matrigel (n 7). After the tumor volume reached about 50mm^3^, 2 × 10^6^ Δ Ecto or EphA2 CAR-NK92 cells were injected five times through intravenous tail vein injection. Tumor size was measured using digital calipers at indicated time points and calculated using the formula length × width^2^ × 0.5. When 32 days after H460 inoculation, the tumor was extracted and tumor weight was measured. A time point was recorded as the time of tumor recurrence when the maximum bioluminescence was higher or the bioluminescent area was larger than at previous time points for a given animal. The CAR-NK cells used in the animal experiments were all obtained from the same tube (batch) of cryopreserved cells, which were continuously cultured and used in the required number of cells.

### 2.11 Tumor infiltration lymphocyte analysis

On day 14 after infusion, three mice per group were sacrificed, and tumor tissues were harvested for tumor infiltration lymphocyte analysis. Tumor tissues were separated into single cells using the tumor dissociation kit (Miltenyi Biotec, Germany), according to manufacturer’s instruction. Single cells of leukocytes were stained with anti-CD3 antibody and analyzed.

### 2.12 Statistical analysis

Data were analyzed using Prism (version 7,0, GraphPad Software) and results were presented as mean ± SD or mean ± SEM. Statistical analyses were performed using two-tailed Student’s t-test or two-way ANOVA test (*p < 0.05, **p < 0.01, ***p < 0.001) for comparison of experimental groups.

## 3 Results

### 3.1 Designing and identifying the EphA2 lentivirus

A human synthetic single-chain variable fragment (scFv) phage display library was used to select an anti-EphA2 scFv antibody that bound to EphA2. The ability of scFv to bind to an EphA2-overexpressing cell line was verified via enzyme-linked immunosorbent assay (ELISA) and FACS analysis to select EphA2-specific clones (**Supplementary Figures 1A, B**). Based on these results, scFv 79 and 85, which exhibited excellent binding abilities, were selected.

**Figure 1:**
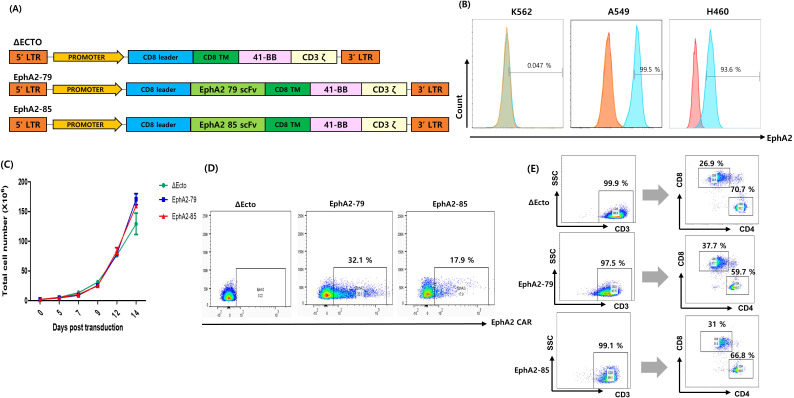
Generation of erythropoietin-producing hepatocellular carcinoma A2 (EphA2) specific T cells and Characterization of Δ Ecto and EphA2 CAR-T cells. **(A)** Design of the Δ Ecto, EphA2-79 and EphA2-85 constructs. **(B)** Expressions of EphA2 (blue line) and Isotype (red line) on K562 (EphA2 MFI ratio; 1.04), A549 (EphA2 MFI ratio; 3.7) and H460 (EphA2 MFI ratio; 1.83) cells were detected by FACS analysis. **(C)** The Δ Ecto and EphA2 CAR-T cell number was counted every 2 day in triplicate using a hematocytometer. Data are expressed as the mean ± SEM of three independent experiments. **(D)** Lentiviral vectors were used to express the EphA2 CAR on activated T cells, and flow cytometry was used to analyze the results. The percentage of EphA2 CAR-T positive cells is shown by the numbers in the panels. **(E)** human T cells are isolated by anti-human CD3 gating and finally the number of human CD8+ and CD4+ T cells are determined.

To develop an effective CAR-T therapeutic agent that targets lung cancer, a lentivirus expressing EphA2 CAR was constructed. The EphA2 scFv, Nos. 79 and 85 were discovered and cloned into a lentiviral vector with CD8 TM, 41-BBB, and CD3 ζ fragments. Δ Ecto was a control group lacking scFv (**Figure 1A**). To confirm the EphA2 expression rate in various cancer cells, K562 and NSCLC cell lines A549 and H460 were analyzed using fluorescence-activated cell sorting (FACS). The results showed that K562 cells did not express EphA2, whereas A549 and H460 cells showed 100% EphA2 expression (**Figure 1B**).

Using Δ Ecto, EphA2-79, and EphA2-85 lentiviruses, CAR was transformed into T cells to produce Δ Ecto, EphA2-79, and EphA2-85 CAR-T cells. Cell proliferation was measured at three-day intervals. T cells were confirmed to have sufficiently expanded to 1 × 10^8^ or more 14 days after transduction (**Figure 1C**). To measure the efficient CAR expression of Δ Ecto, EphA2-79, and EphA2-85 CAR-T cells, FACS through FITC-conjugated recombinant human EphA2 was used. EphA2-79 and EphA2-85 CAR-T cells stably expressed CAR compared to the control Δ Ecto CAR-T cells, with expression rates of approximately 32% and 18%, respectively (**Figure 1D**). The CD4 and CD8 marker subsets of CAR-T cells were confirmed after CD3^+^ cell gating. CD4^+^ and CD8^+^ of Δ Ecto CAR-T cells were 70.7%, 26.9%, those of EphA2-79 were 59.7%, 37.7%, and those of EphA2-85 were 66.8%, and 31%, respectively (**Figure 1E**). When CD3 was expressed at a rate of more than 95%, almost all cells differentiated into T cells, and CAR expression was effectively confirmed in the transformed CAR-T cells.

### 3.2 EphA2-specific T cells kill EphA2 target cells and induce cytokine release

**Figure 2:**
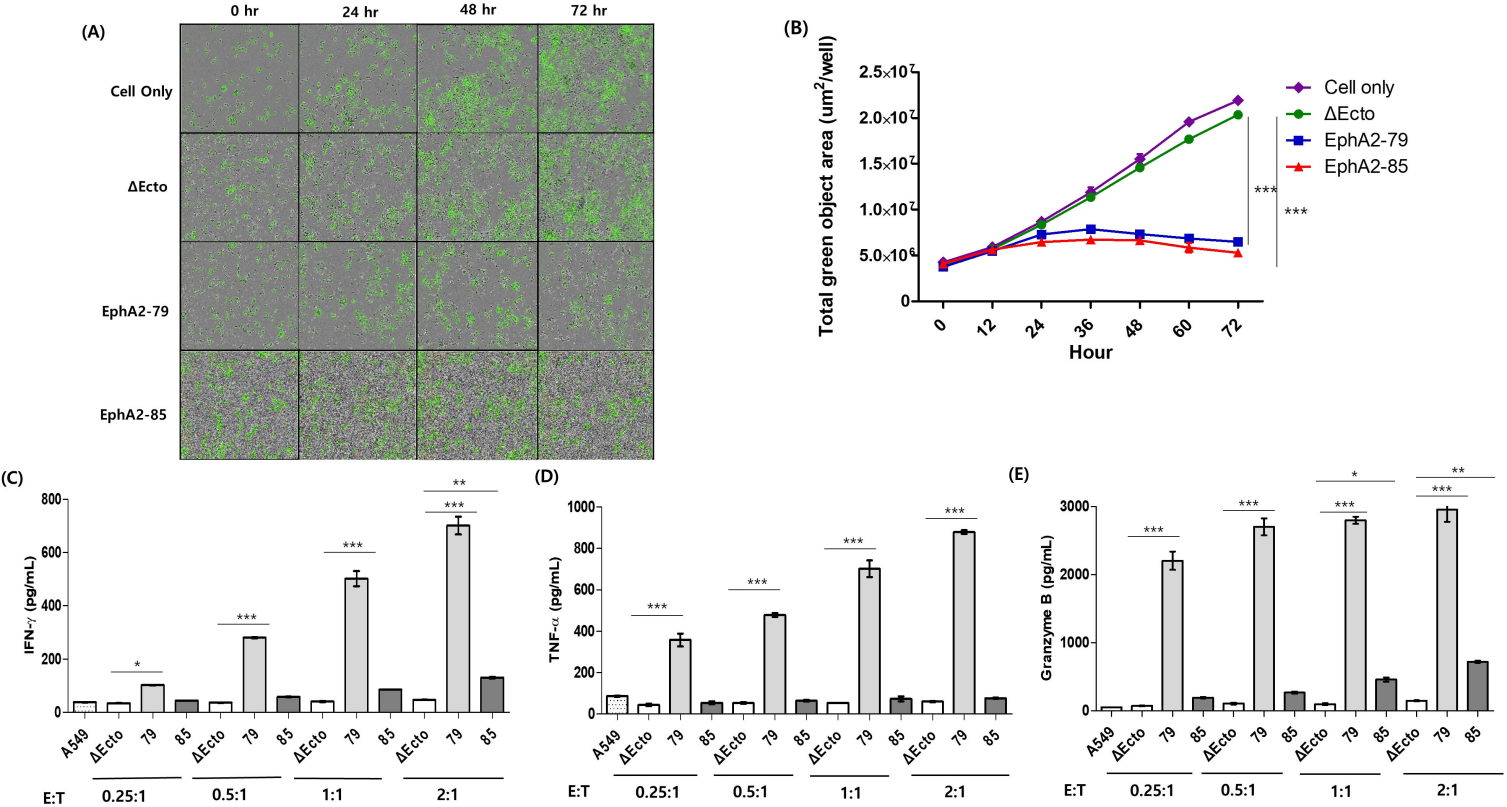
The cytotoxicity and cytokine secretion of EphA2 CAR-T cells. **(A)** The GFP signal of A549 cells were observed using a fluorescence microscope in real time. **(B)** A graph shows how the overall GFP signal has increased over time in a 1:1 ratio. The blue line represents EphA2-79 CAR-T while the red line is EphA2-85 CAR-T. **(C)** IFN-γ, **(D)** TNF-α, and **(E)** Granzyme B concentrations in cell culture supernatants were calculated by ELISA. Four different effector to target cell ratios were used when cultivating Δ Ecto or EphA2 CAR-T cells with tumor cells for 16 hours. Results are representative of three independent experiments. Data are expressed as the mean ± SEM of three independent experiments ***p < 0.001; **p < 0.01; *p < 0.05.

A549, an NSCLC cell line with 100% EphA2 expression, was selected as the target cell line, and an *in vitro* efficacy evaluation was conducted. The cytotoxicity of Δ Ecto, EphA2-79, and EphA2-85 CAR-T cells for the target A549 cell were confirmed in real-time. A549 cells expressing green fluorescence protein (GFP) were prepared and used in this study. Real-time data were obtained for up to 72 h at 12-h intervals by coculturing the ratio of effector cells to target cells at 1:1 (**Figure 2A**). The change in the GFP signal of cells incorporated with CAR-T cells was calculated and no difference was observed in GFP signal change between the control group Δ Ecto CAR-T and EphA2-79 and EphA2-85 CAR-T cells until 12 h. However, as time passed, the GFP signals of EphA2-79 and EphA2-85 CAR-T cells decreased significantly compared to the control group Δ Ecto CAR-T cells. EphA2-79 and EphA2-85 CAR-T cells recognized EphA2 and killed the target cancer cells (**Figure 2B**). GFP fluorescence photographs taken in real time at 12-h intervals and GFP signals until 72 h are presented as graphs.

Δ Ecto, EphA2-79, and EphA2-85 CAR-T cells, and target cancer cell lines were cocultured to measure interferon (IFN)-γ, tumor necrosis factor (TNF)-α, and Granzyme B, cytokines secreted via killing target cancer cells, using ELISA. This was confirmed using a culture medium cocultured for 16 h with CAR-T cells, and lung cancer cells at various ratios. For EphA2-79 CAR-T cells, the secretion of all cytokines significantly increased as the proportion of CAR-T cells increased. In contrast, for EphA2-85 CAR-T cells, only IFN-γ and Granzyme B showed statistically significant values and increased at the 2:1 ratio; however, only a slight increase, without statistical significances, were observed in the remaining cells (**Figures 2C–E**). Overall, EphA2-79 and EphA2-85 CAR-T cells efficiently recognized and killed EphA2 in target cancer cells *in vitro*. Correspondingly, sufficient cytokine secretion was induced as the proportion of CAR-T cells increased.

### 3.3 EphA2 CAR-T cells show increased antitumor efficacy *in vivo*

**Figure 3:**
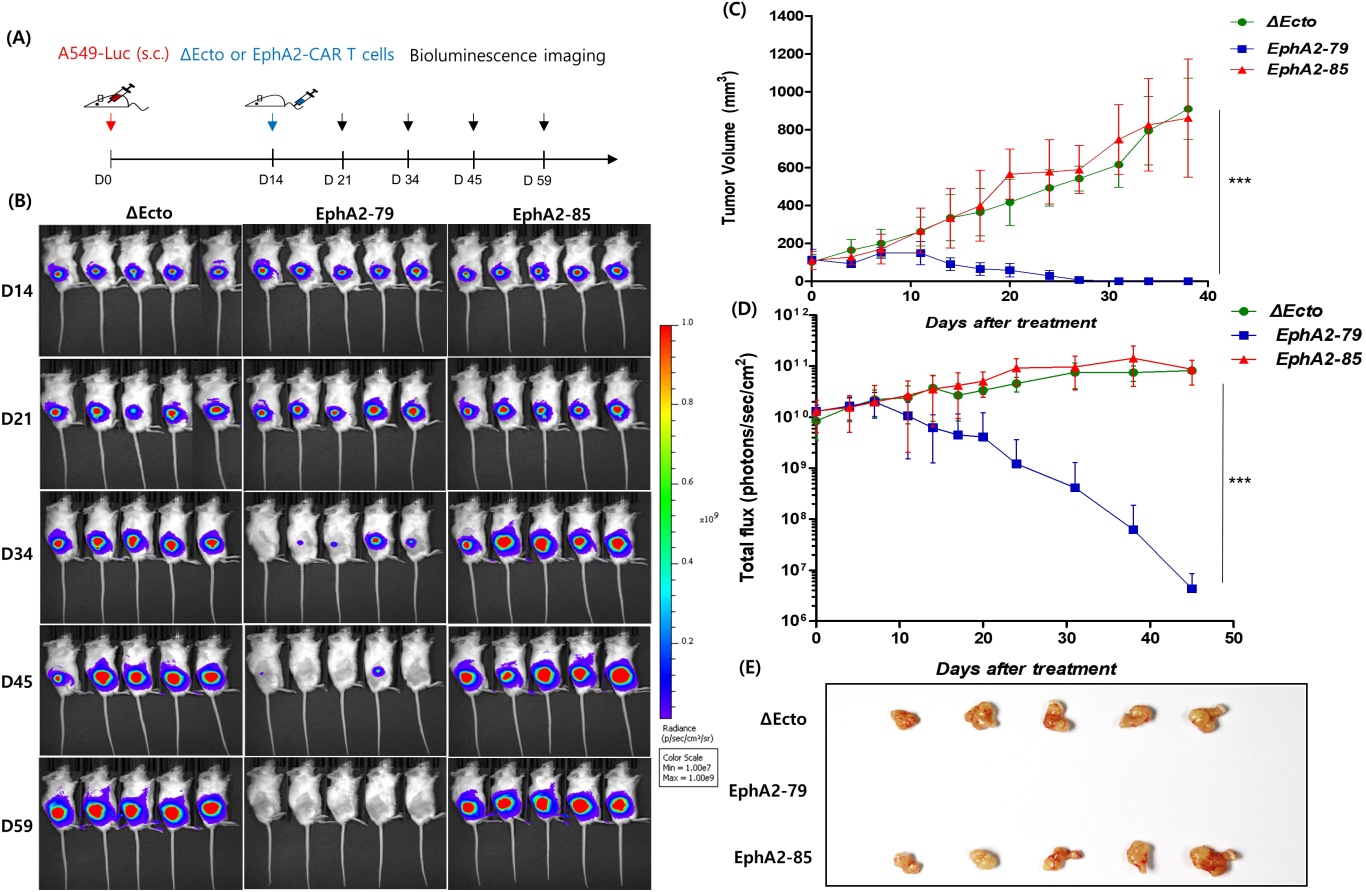
EphA2 CAR-T cells exhibit strong antitumor efficacy against A549 in xenograft studies **(A)** Illustration of the animal experiment in schematic form. **(B)** Bioluminescence images displaying the tissue in various mice groups on days 14, 21, 34, 45, and 59. **(C)** Every three days, the tumor volume dynamic curves were measured. **(D)** Measurement of the overall flux (photon/s) from the entire body region, excluding the tail. The blue line represents EphA2-79 CAR-T, and the red line is EphA2-85 CAR-T. **(E)** A tumor formation assay was performed after administering chimeric antigen receptor (CAR)-T cells over 59 days. Three different experiments were performed with comparable outcomes. ***p < 0.001.

To confirm the *in vivo* efficacy of EphA2 CAR-T cells, an A549 cell line expressing luciferase (A549-Luc) was prepared and injected at a dose of 1 × 10^6^ cells into the subcutaneous area of six-week-old NOG mice. When the subcutaneous tumor size reached 200 mm^3^ after 14 days, CAR-T cells were intravenously injected at a density of 5 × 10^6^ cells/mouse (**Figure 3A**). Tumor size, weight, and bioluminesce imaging were measured at intervals of three days to confirm the antitumor effects. EphA2-79 CAR-T cells showed a continuous decrease in tumor size after 11 days, and with the tumor disappearing entirely by the end of the test. In contrast, Δ Ecto and EphA2-85 CAR-T cells exhibited a trend of increasing tumor size (**Figures 3B–D**). The weight of the group injected with EphA2-79 CAR-T cells decreased sharply on day 4 but recovered quickly, showing no abnormal changes until the end of the test. No significant weight change was observed in the other group throughout the test. An autopsy was performed 45 days after CAR-T cell injection. However, the results for the EphA2-79 group could not be obtained because the tumors had disappeared on day 40. The Δ Ecto and EphA2-85 CAR-T cell groups separated the tumor, which was confirmed using a photograph (**Figure 3E**).

**Figure 4:**
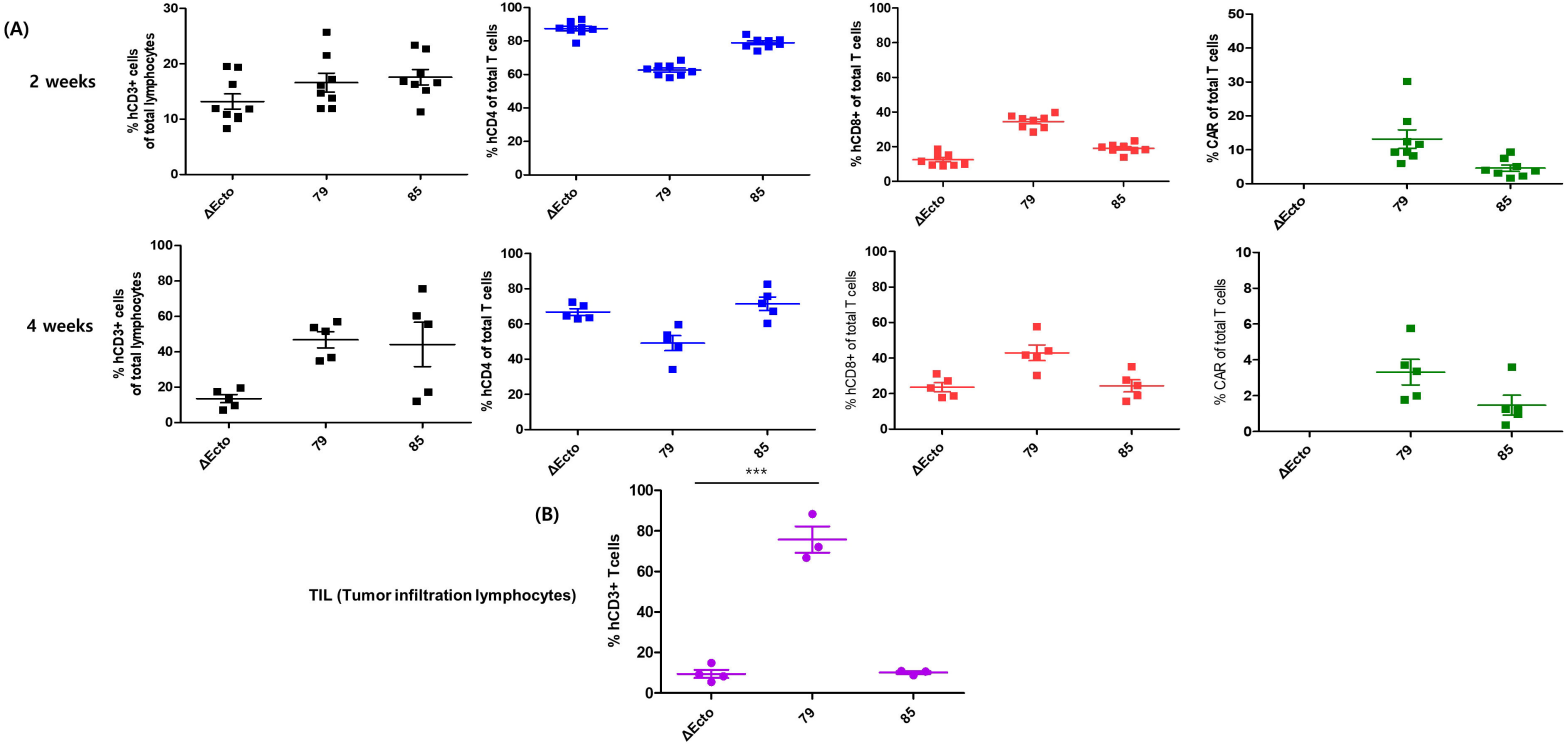
Phenotypic characterization of peripheral blood T cell subsets and tumor-infiltrating lymphocytes (TILs) following treatment with EphA2 CAR-T cells. **(A)** Peripheral blood samples were collected 2 and 4 weeks after infusion in the xenograft model. Lymphocytes were isolated from peripheral blood and analyzed via FACS using various antibodies to determine the percentages of CD3+, CD4+, CD8+, and CAR-positive cells. **(B)** Tumors were harvested from mice, and T cells were isolated from the tumor tissue. The collected cells were stained with anti-CD3 antibodies to confirm T cell infiltration. EphA2-79 CAR-T cell treatment resulted in a significant increase in CD3+ T cell infiltration within mouse tumors. ***p < 0.001.

After injecting the CAR-T cells, mouse blood was collected on days 14 and 28 to observe T cell proliferation and subtype changes in the blood, and T cell phenotype and CAR expression were confirmed. In the second week, CD3 expression in all three groups was approximately 20%, and CAR expression rates were approximately 15% and 5% in EphA2-79 and EphA2-85 CAR-T cells, respectively (**Figure 4A** upper). In all three groups, CD3 and CD8 expression levels were higher at week 4 than at week 2. CAR expression was significantly reduced in EphA2-79 and EphA2-85 CAR-T cells compared with that at week 2 (**Figure 4A** lower). In addition, the tumors were isolated. CD3 markers were analyzed to determine CAR-T cell penetration into the tumors. Approximately 80% CD3 expression was observed in EphA2-79 CAR-T cells, whereas less than 10% was expressed in the other groups. Significant results were obtained when compared with the control group Δ Ecto and EphA2-85 CAR-T cells (**Figure 4B**).

### 3.4 EphA2 CAR-NK92 cells also exhibited killing activity both *in vitro* and *in vivo*

**Figure 5:**
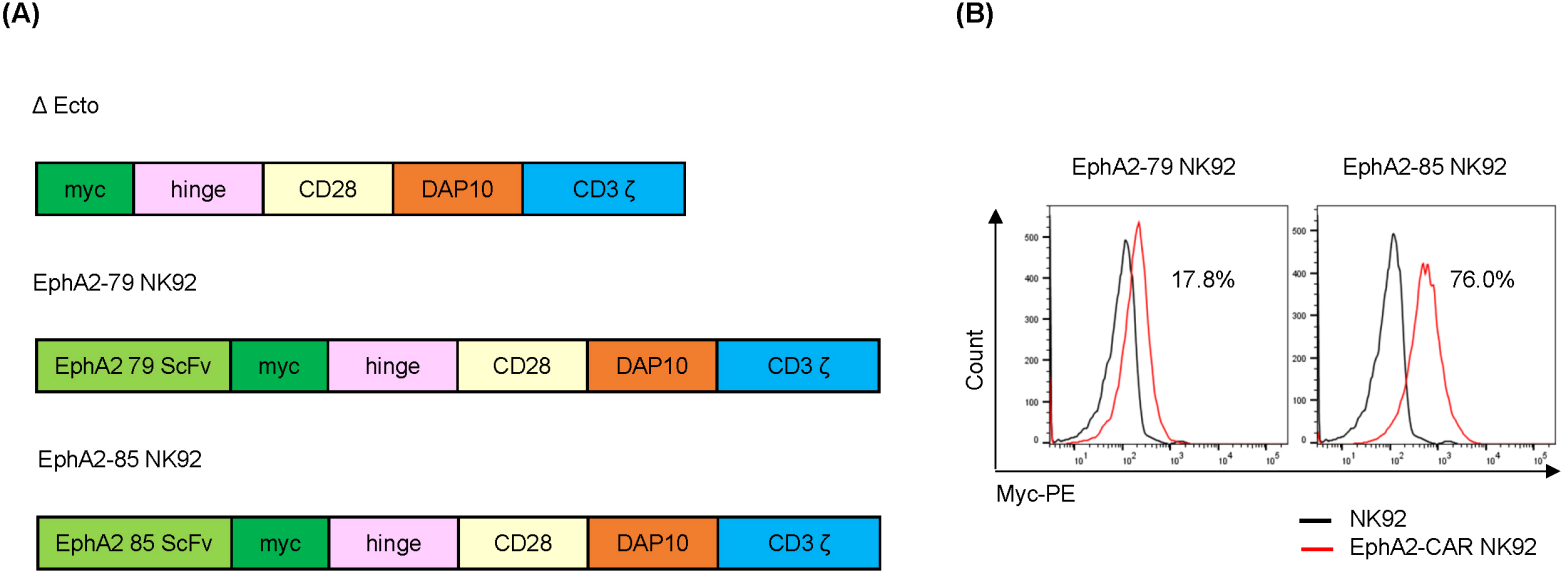
Construction of EphA2-CAR NK92 cells **(A)** Construction of EphA2-CAR NK92 system (EphA2 scFv clone: 79, 85). **(B)** Chimeric antigen receptor (CAR) level expressed on the NK92 or EphA2-CAR NK92 cell surface. EphA2-CAR NK92 cells were selected via transduction of EphA2-CAR lentivirus into NK92 cells and then treated with puromycin for three days. CAR expression was analyzed via flow cytometry (FACS) using a Myc-PE conjugated antibody.

The EphA2 CAR-NK92 system is composed of three ecto-domains (comprising scFv against EphA2; clones 79, and 85, respectively, Myc as a CAR expression identifier, and a CD8-based hinge as a spacer in immune synapse formation), a transmembrane domain originating from CD28, and an endo-domain with cytoplasmic regions of CD28, Dap10, and CD3ζ (**Figure 5A**). To establish EphA2 CAR-NK92 cells, the EphA2 CAR was introduced into NK92 cells using a lentiviral vector. Transduced NK92 cells were selected using puromycin after three days. The 79 and 85 EphA2 CAR-NK92 cells expressed 17.8% and 76.0% of CAR, respectively, compared to the parental NK92 cells (**Figure 5B**).

**Figure 6:**
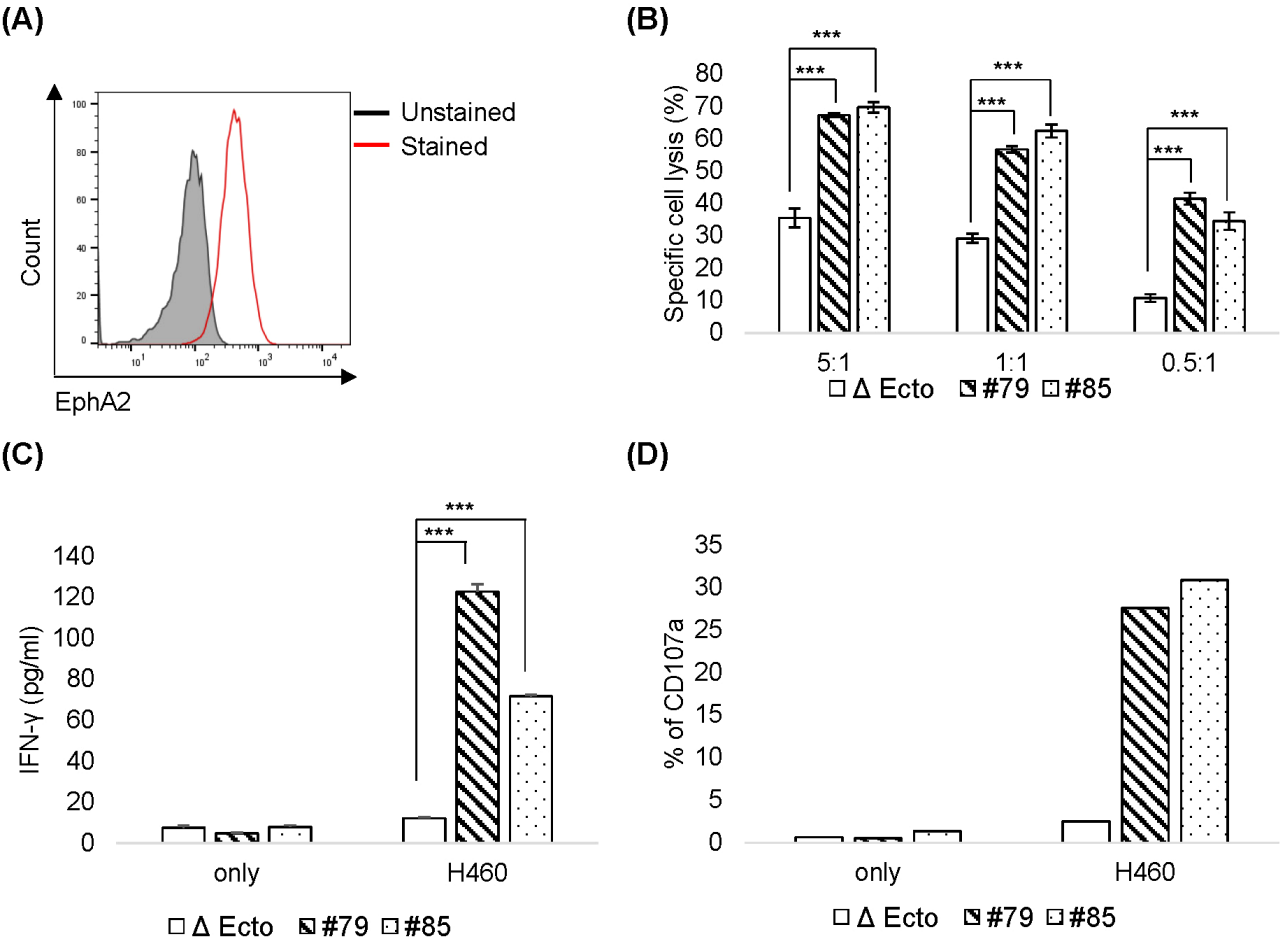
Cytotoxicity of EphA2 CAR-NK92 cells against H460 **(A)** EphA2 expression levels in H460 cells were confirmed through fluorescence-activated cell sorting analysis. **(B)** H460 cell killing activity by Δ Ecto or EphA2 CAR-NK92 cells (#79, #85) was measured via calcein AM-based cytotoxicity assay at the indicated natural killer (NK): H460 (E: T) ratio. **(C, D)** Interferon (IFN)-γ secretion **(C)** and CD107a expression **(D)** in EphA2 CAR-NK92 cells cultured with H460. Each value represents the percentage of CD56^+^CD107a^+^ cells in flow cytometric density plots. Error bars for **(B, C)** are ± s.d. based on technical triplicate measurements. Experiments were repeated twice independently and showed similar results. Statistical significance was determined using the Student’s *t*-test: ***p < 0.001.

To assess CAR activity against target tumors, EphA2 CAR-NK92 cells were cocultured *in vitro* with three tumor cell lines: H460 (human lung cancer), MDA-MB-231 (human breast cancer), and K562 (Chronic Myelogenous Leukemia). EphA2 surface expression was evaluated using flow cytometry. H460 (**Figure 6A**) and MDA-MB-231 expressed EphA2, whereas K562 cells did not express it (**Supplementary Figure 2A**). To evaluate the cytotoxicity, NK92 and EphA2 CAR-NK92 cells were cocultured with H460, MDA-MB-231, and K562 cells. The cytotoxicity of EphA2 CAR-NK92 cells was higher than that of NK92 cells in H460 (**Figure 6B**) and MDA-MB-231 cells, but was not different in K562 cells (**Supplementary Figure 2B**). EphA2 CAR NK cells were cocultured with H460, MDA-MB-231, or K562 cells to evaluate cytokine secretion and degranulation. IFN-γ section and degranulation marker CD107a expression were increased in EphA2 CAR-NK92 (**Figures 6C, D**, **Supplementary Figures 2C, D**). These results indicate that EphA2 CAR-NK92 cells exhibit ligand-dependent cytotoxicity against tumor cells.

**Figure 7:**
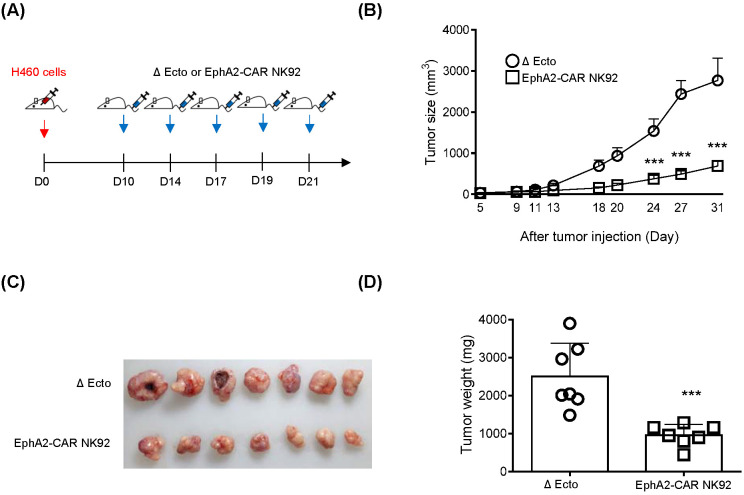
EphA2 CAR-NK (#79) cells display enhanced killing effects in H460 xenograft mouse model. **(A)** The H460 xenograft mouse experimental scheme. Mice were subcutaneously inoculated at the flanks of mice with 50% Matrigel solution. And then Δ Ecto or EphA2-CAR NK92 (#79) cells were intravenously injected five times (n: 7). **(B)** Tumor volume was measured using digital calipers at indicated time points. **(C, D)** Tumor image **(C)** and tumor weight **(D)** from both groups are shown at 32 days after H460 inoculation. Error bars for **(B, D)** represent ± SEM. Experiments were repeated twice independently and showed similar results each other. Significance was determined using the Student’s *t*-tests **(B, D)** or Mann-Whitney U **(C)**: ^***^p < 0.001.

As H460 cells expressed EphA2 and EphA2 CAR-NK92 cell cytotoxicity was higher than that of NK92 in H460 cells (**Figure 6**), we investigated the efficacy of EphA2 CAR-NK92 cells in solid tumors using an H460 xenograft mouse model. To establish a xenograft model, 3 × 10^6^ H460 cells were subcutaneously injected into the flanks of mice. After 10 days, Δ Ecto or EphA2 CAR-NK92 cells were injected intravenously five times (on day 10, 14, 17, 19, 21) (**Figure 7A**). Based on the results of the *in vivo* experiment using EphA2 CAR-T cells, we used EphA2 CAR-NK cells with EphA2 scFv #79. The results showed that solid tumor growth and volume reduced in mice injected with EphA2 CAR-NK92 cells compared to that in Δ Ecto cells (**Figures 7B–D**). Through *in vitro* and *in vivo* assays, we confirmed that the killing efficacy of EphA2 CAR NK cells was greater than that of Δ Ecto cells.

## 4 Discussion

This study showed that CAR-T/NK cells targeting EphA2 have anticancer effects against A549 and H460 lung cancer cell lines *in vitro* and *in vivo*. Compared to normal T cells, EphA2 CAR-T cells showed higher killing efficacy against A549 cells, and increased IFN-γ, TNF-α, and Granzyme B secretion. In addition, the xenograft mouse model confirmed that A549 tumor growth was strongly inhibited, and the number of tumor-infiltrating cells increased. These anticancer effects were confirmed not only in T cells but also in NK cells. We confirmed that EphA2 CAR NK cells have a high killing capacity for H460 cells and effectively suppress tumor growth. Thus, these results show that CAR-T/NK cells targeting EphA2 may represent a more effective immune cell therapy for lung cancer, particularly for NSCLC with high EphA2 expression.

We discovered a novel anti-EphA2 scFv from a fully human antibody library, generated CAR-T/NK cells, and confirmed its antitumor activity. CAR-T/NK cells made with fully humanized EphA2 scFv have reduced immunogenicity compared to cells without, which may increase the treatment’s safety and antitumor efficacy. In addition, their high specificity may reduce their toxicity to non-target cells and minimize side effects. These positive results may provide potential advantages for increasing clinical trial success (19, 20).

Several EphA2-specific CAR-T cells have been developed and evaluated. They have been reported to show high anticancer efficacy against glioblastomas (21, 22) and esophageal squamous cell carcinomas (23). Additionally, EphA2 CAR-T cells targeting NSCLC have been reported (18). Although we did not perform direct comparative experiments with our EphA2 CAR-T cells and could not make an exact efficacy comparison, unlike in previously reported studies, we discovered a novel anti-EphA2 scFv from a fully human antibody library and utilized it in CAR-NK to confirm its high anticancer efficacy. For EphA2 CAR-NK cells, the development and validation of EphA2 CAR-NK cells targeting NSCLC have not yet been reported.

CAR-T cell therapy is highly effective in treating blood cancers but is limited by severe side effects, such as CRS, neurotoxicity, and high costs (4). To address these challenges, we explored CAR-NK cell therapy, which offers advantages like a lower risk of CRS and GvHD (24), rapid activation, and potential off-the-shelf availability (25, 26). Despite its benefits, CAR-NK therapy faces limitations, including reduced *in vivo* expansion and persistence (27). Combining CAR-T and CAR-NK cells could overcome these challenges, leveraging CAR-T cells’ precise antigen targeting and robust expansion alongside CAR-NK cells’ broad immune response (26), which addresses tumor heterogeneity and antigen escape (27). Additionally, CAR-NK cells can initiate early treatment and modulate the tumor microenvironment (28), enhancing CAR-T efficacy in solid tumors. Our findings suggest that by harnessing these complementary strengths, combination therapies have the potential to significantly improve both immediate and long-term treatment outcomes, highlighting the promise of this approach in future cancer immunotherapy development.

While this study provides evidence of the efficacy of EphA2 CAR-T/NK cells against EphA2-expressing tumor cells, certain limitations must be acknowledged. The combined therapeutic potential of CAR-T and CAR-NK cells was not directly evaluated, and the long-term safety and efficacy of CAR-NK cells remain to be established. Future studies should investigate the synergistic effects of CAR-T and CAR-NK cells in coculture systems and *in vivo* models to determine their potential as combination therapies. Additionally, further exploration of CAR-T/NK cells in a broader range of EphA2-positive cancer models is warranted to validate their clinical applicability.

In conclusion, the results of this study suggest that CAR-T/NK cells targeting EphA2 represent a promising therapeutic strategy for NSCLC, particularly for tumors with high EphA2 expression. This approach may help address the limitations of current CAR cell therapies, paving the way for safer and more effective treatments for lung cancer.

## Data Availability

The original contributions presented in the study are included in the article/**Supplementary Material**. Further inquiries can be directed to the corresponding authors.
