# Supplementary material for: Developing CAR-T/NK cells that target EphA2 for non-small cell lung cancer treatment
Source: Front Immunol. 2025 Mar 13;16:1448438. doi: 10.3389/fimmu.2025.1448438 (PMC11966065; doi:10.3389/fimmu.2025.1448438)
Supplement: Supplementary file 1 [file DataSheet1.docx]

Developing CAR-T/NK cells that target EphA2 for non-small cell lung cancer treatment

Seok Min Kim^1, †^, Soo Yun Lee^1, †^, Seo In Kim^2, 3, †^, Ji Yeong Bae^2, 3^, Jin Tae Hong^3^, Seona Jo^1, 4^, Ji Hyun Kim^1, 4^, Hyo-Young Chung^2,^ * and Tae-Don Kim^1, 4,^ *

^1^Center for Gene & Cell Therapy, Korea Research Institute of Bioscience and Biotechnology (KRIBB), 125 Gwahak-ro, Yuseong-gu, Daejeon 34141, Republic of Korea

^2^New Drug Development Center, Osong Medical Innovation Foundation, 123, Osongsaengmyeong-ro, Osong-eup, Cheongju-si, Chungbuk 28160, Republic of Korea

^3^College of Pharmacy and Medical Research Center, Chungbuk National University, 194-31, Osongsaengmyeong 1-ro, Osong-eup, Cheongju-si, Chungbuk 28160, Republic of Korea

^4^Department of Functional Genomics, KRIBB School of Bioscience, Korea University of Science and Technology (UST), 217 Gajeong-ro, Yuseong-gu, Daejeon 34113, Republic of Korea

^†^ These authors contributed equally

* Correspondence: hchung@kbiohealth.kr (H.Y.C) and tdkim@kribb.re.kr (T.D.K)

Keywords: Cell therapy, Chimeric antigen receptor (CAR), CAR-T cell, CAR-NK cell, erythropoietin-producing hepatocellular carcinoma A2 (EphA2), non-small cell lung cancer (NSCLC),

## Supplementary Figures


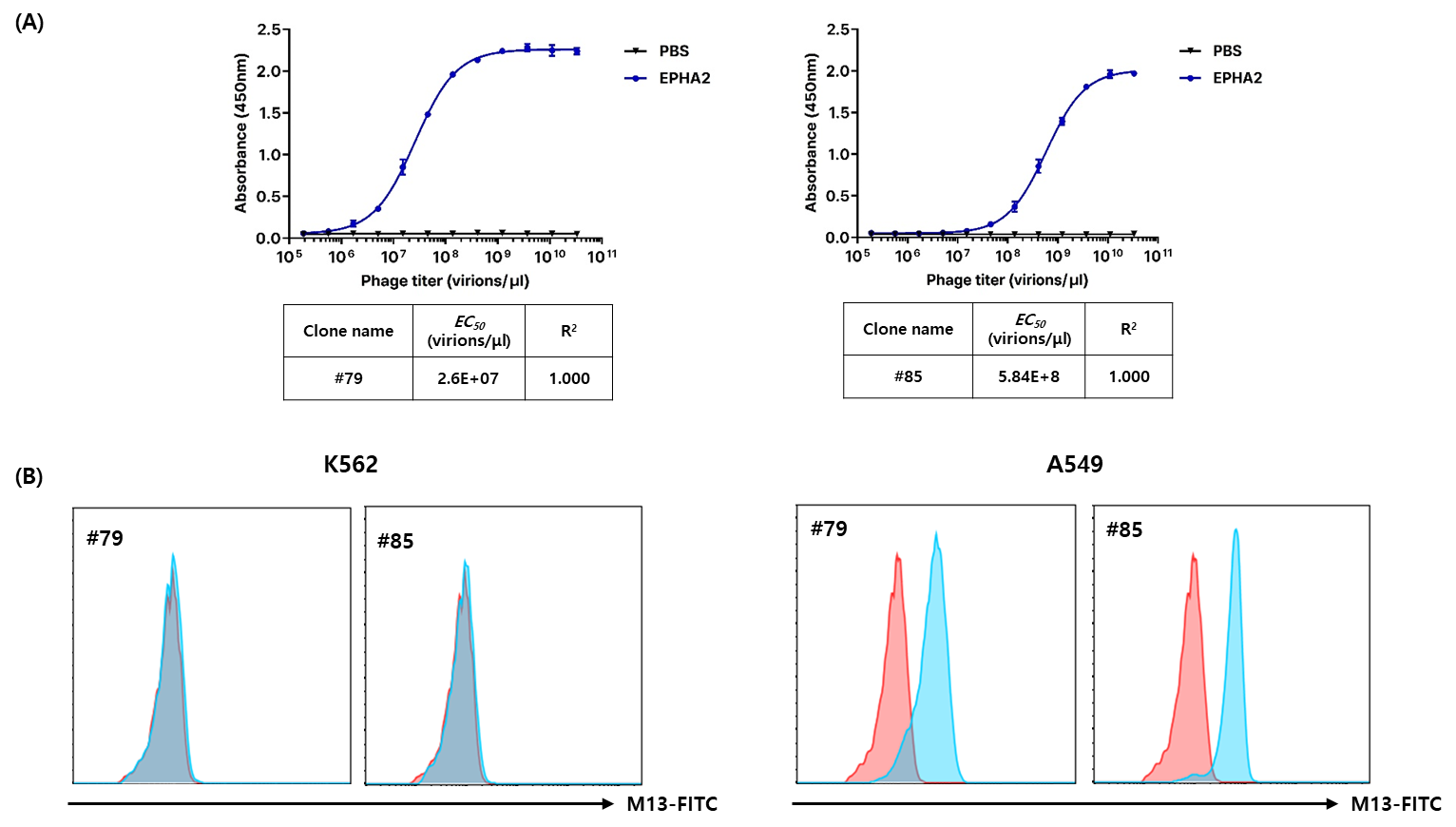


**Supplementary Figure 1. ELISA assay and flow cytometry analysis of antigen binding for the selected EphA2 scFvs.** (A) The #79 and #85 scFvs were displayed on phage particles and amplified through phage amplification. After determining the phage titer, serial dilutions of the phage were prepared, and an ELISA assay was performed to evaluate the antigen-binding ability of the selected clones. The results confirmed that both scFvs bind specifically to EphA2. (B) K562 and A549 cells were treated with the selected scFvs (#79 and #85) and subsequently stained with conjugated anti-M13 FITC for flow cytometry analysis. K562 cells, which are negative for EphA2 expression, served as a negative control, while A549 cells, which are positive for EphA2 expression, served as a positive control. The red line represents the negative control, and the blue line represents the scFv antibody.


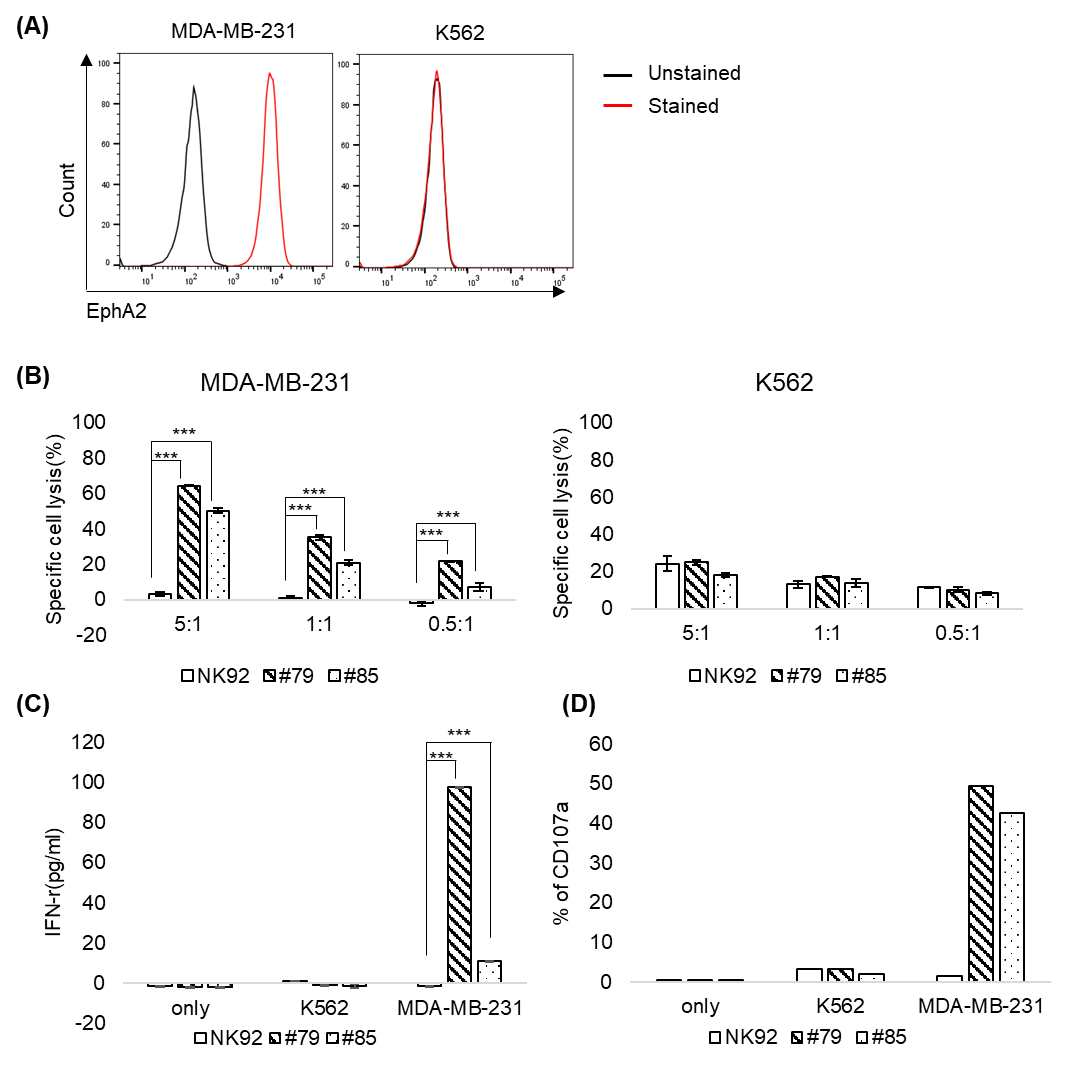


**Supplementary Figure 2. Cytotoxicity of EphA2-CAR NK92 cells against MDA-MB-231 and K562 (A)** EphA2 expression levels in cancer cell lines MDA-MB-231 and K562 were confirmed using fluorescence activated cell sorting analysis. **(B)** Cytotoxicity of EphA2-CAR NK92 cells toward target cells (Left, MDA-MB-231; Right, K562) according to EphA2 expression. NK92 or EphA2-CAR NK92 cells were co-cultured with calcein-stained tumor cells at E:T ratios of 0.5:1, 1:1, and 0.5:1 for 4 h. **(C–D)** Interferon (IFN)-γ secretion **(C)** and CD107a expression **(D)** in EphA2-CAR NK92 cells cultured with tumor cells expressing or not expressing EphA2. Each value represents the percentage of CD56^+^CD107a^+^ cells in the flow cytometry density plots. Error bars for panel B, C and D are ± s.d. based on technical triplicate measurements. The experiments were independently repeated twice and showed similar results. Statistical significance was determined using Student’s *t*-test: ***p < 0.001; **p < 0.01; *p < 0.05.
